# Hybrid Membranes of PLLA/Collagen for Bone Tissue Engineering: A Comparative Study of Scaffold Production Techniques for Optimal Mechanical Properties and Osteoinduction Ability

**DOI:** 10.3390/ma8020408

**Published:** 2015-01-26

**Authors:** Flávia Gonçalves, Ricardo Bentini, Mariana C. Burrows, Ana C. O. Carreira, Patricia M. Kossugue, Mari C. Sogayar, Luiz H. Catalani

**Affiliations:** 1 Instituto de Química, Universidade de São Paulo, Av. Prof. Lineu Prestes, 748, 05508-000 São Paulo, SP, Brasil; E-Mails: fgoncalves@usp.br (F.G.); ricardobentini@usp.br (R.B.); marianacarvalho@usp.br (M.C.B.); anaclaudiaoli@gmail.com (A.C.O.C.); mcsoga@iq.usp.br (M.C.S.); 2 Faculdade de Medicina, Núcleo de Terapia Celular e Molecular (NUCEL)—Núcleo de Estudos e Terapia Celular e Molecular (NETCEM), Universidade de São Paulo, Rua Pangaré 100, 05360-130 São Paulo, SP, Brasil; E-Mail: pmkossugue@gmail.com

**Keywords:** electrospinning, scaffolds, collagen, synthetic polymer

## Abstract

Synthetic and natural polymer association is a promising tool in tissue engineering. The aim of this study was to compare five methodologies for producing hybrid scaffolds for cell culture using poly-l-lactide (PLLA) and collagen: functionalization of PLLA electrospun by (1) dialkylamine and collagen immobilization with glutaraldehyde and by (2) hydrolysis and collagen immobilization with carbodiimide chemistry; (3) co-electrospinning of PLLA/chloroform and collagen/hexafluoropropanol (HFP) solutions; (4) co-electrospinning of PLLA/chloroform and collagen/acetic acid solutions and (5) electrospinning of a co-solution of PLLA and collagen using HFP. These materials were evaluated based on their morphology, mechanical properties, ability to induce cell proliferation and alkaline phosphatase activity upon submission of mesenchymal stem cells to basal or osteoblastic differentiation medium (ODM). Methods (1) and (2) resulted in a decrease in mechanical properties, whereas methods (3), (4) and (5) resulted in materials of higher tensile strength and osteogenic differentiation. Materials yielded by methods (2), (3) and (5) promoted osteoinduction even in the absence of ODM. The results indicate that the scaffold based on the PLLA/collagen blend exhibited optimal mechanical properties and the highest capacity for osteodifferentiation and was the best choice for collagen incorporation into PLLA in bone repair applications.

## 1. Introduction

The electrospinning process was patented in 1934 [1], and its use for cell scaffold design is widely accepted and explored in various scientific fields [2,3]. The process for obtaining electrospun materials consists of establishing a potential difference between the polymeric solution or melted polymer and the collector, creating an electric field between them and ejecting the electrically charged polymeric solution onto the collector. The electric field stretches the polymer chains, and the polymer is randomly deposited on the collector [2].

Electrospun materials can be produced from synthetic or natural polymers [3]. Among the synthetic polymers that can be used, aliphatic polyester poly-l-lactide (PLLA) excels as a biomaterial because of its semi-crystalline form, which enables the production of high tensile strength [4], biocompatible, easily processed materials with satisfactory mechanical properties for bone regeneration [4]. However, PLLA does not have specific binding sites for cell adhesion and is highly hydrophobic, causing poor interaction with cells and precluding the flow of nutrients [5,6].

Among the natural polymers used as biomaterials, collagen is prominent. As a protein formed mainly of glycine, proline and hydroxyproline, collagen exhibits a highly organized structure [7]. Collagen is the primary extracellular matrix protein of several tissues, such as bone and conjunctive tissue, exhibiting specific sites for cell adhesion and good compatibility [8]. However, the use of collagen presents some limitations, such as a high degradation rate, low processability and inadequate mechanical properties for bone-related applications [9,10]. Hence, the concomitant use of synthetic and natural materials, such as PLLA and collagen, has proven to be an excellent alternative for combining the positive aspects of both materials [5,11].

Depending on the conditions used, the electrospinning of collagen solutions may result in fiber mats with a diameter near that of natural collagen fibrils [12]. However, the main solvents used to dissolve collagen, such as fluorinated alcohols 1,1,1,3,3,3-hexafluoro-2-propanol (HFP) or 2,2,2-trifluoroethanol (TFE), also destabilize the hydrogen bonding of collagen’s triple-helix structure, leading to denaturation [13]. A circular dichroism study reported the denaturation of 45% of the triple-helix structure in an 8% wt/vol collagen-HFP solution [12]. Using gel electrophoresis, analyses of gelatin and collagen bands when solubilized in HFP indicated a 58% loss of collagen. After electrospinning, the total loss increased to 68%. These values increased to 93% and 99.5%, respectively, when submitted to pepsin degradation. Pepsin is not known to degrade intact structures of collagen’s triple-helix backbone, but it is known to be active on modified structures, providing evidence of collagen denaturation [13]. Other authors have suggested that the use of acetic acid as a solvent minimizes the degradation of native collagen structures. However, more studies are required to understand the exact effect of acetic acid as a collagen solvent because only a small advantage is observed with respect to HFP use [14].

One approach to avoid electrospinning-related problems is through the immobilization of collagen onto a polymer matrix surface, e.g., PLLA, which has been demonstrated to be an excellent alternative for increasing hydrophilicity and thus cell adhesion and proliferation while maintaining cell functionality [5,11]. Immobilization can be achieved by anchoring amine or carboxyl groups onto polymer surfaces previously generated through, for example, aminolysis or hydrolysis [15,16]. Although these methodologies have been successfully employed and intensively studied in films and sintered three-dimensional (3D) matrices [17], their use in electrospinning has rarely been reported [18,19]. Electrospun PLLA fiber aminolysis, followed by chitosan immobilization, resulted in increased fibroblast proliferation while maintaining the mechanical properties of the fibers [18]. Accordingly, the attachment of fibronectin to PLLA fibers through aminolysis led to increased epithelial cell proliferation and collagen type IV expression [19].

The aim of the present study was to evaluate and compare different types of collagen associations to PLLA in electrospun fibers. The methodologies used were as follows: (i) electrospinning of co-solutions, resulting in blends; (ii) electrospinning of PLLA followed by collagen immobilization onto the surfaces of fibers; and (iii) co-electrospinning of PLLA and collagen isolated solutions, resulting in hybrid mats. Various solvents and immobilization techniques were quantitatively and qualitatively evaluated and compared.

## 2. Results and Discussion

### 2.1. Generation and Characterization of Scaffolds

Aggregating biological components and synthetic polymers to mimic extracellular matrix (ECM) properties is a widespread technique [5,20]. The use of collagen as the bio-component has resulted in numerous successful examples of such mimicry, most of which have been constructed from collagen-decorated surfaces, in which the amount of collagen is usually scant [20]. Alternatively, blending both components through co-dissolution, followed by film or fiber processing, can yield materials with higher proportions of collagen. Depending on the polymer-to-collagen ratio, the electrospinning of a co-solution may render hybrid materials with fiber populations of different compositions. Co-electrospinning represents the extreme case of this situation, in which two opposed jets of distinct solutions from isolated components are aimed at the same rotating target.

Herein, five different approaches for incorporating a large or reduced amount of collagen into PLLA electrospun scaffolds are compared. Two of these methods are based on collagen immobilization on electrospun PLLA surfaces after modification through aminolysis or hydrolysis. The two other methods involve the co-electrospinning of PLLA/chloroform/DMF (9:1) and collagen/HFP or collagen/acetic acid solutions. Finally, a PLLA/collagen blend was generated from a co-solution in HFP.

An advantage of collagen immobilization onto PLLA is lower degradation and denaturation because collagen is solubilized in an acetic acid solution (4%) [21]. However, as demonstrated below, disadvantages of this method are the following: (i) only small amounts of collagen can be incorporated into the matrix; (ii) fibers must be functionalized prior to processing; and (iii) the scaffold mechanical properties decrease.

Aminolysis (using alkanediamines) and hydrolysis (using basic aqueous solutions) generate respective amino (–NH_2_) and carboxyl (–COOH) groups on the polymer surface. These reactions are dependent on several parameters, such as (i) solvent; (ii) amine type and concentration; (iii) temperature; and (iv) total reaction time [16]. In this study, milder conditions were selected and compared with those used for films and 3D scaffolds [16,17,22]. In all cases, a decrease or complete loss of polymer crystallinity during electrospinning has been observed [23], which could directly affect the material properties and reactivity, thus affecting aminolysis and hydrolysis reaction rates. Additionally, the electrospinning technique is susceptible to the effects of several variables (e.g., polymer concentration, solvent type, flow rate, voltage and needle-collector plate distance) [3], which might govern the diameter of the fibers and indirectly affect the scaffolds’ response to aminolysis and hydrolysis conditions.

Although Chen *et al.* [15] reported the differences in electrospun PLLA fiber hydrolysis using a 0.5 M NaOH solution for 5, 10 and 30 min, we determined that the limiting condition before extensive scaffold degradation was 0.1 M NaOH solution for 3 min. The statistically identical values of –COOH concentration in Table 1 for reaction times of >3 min indicate that hydrolysis reaches a maximum at the surface while continuously leaching to the inner volume of the fibers, compromising the mechanical properties of the fibers instead of creating more reactive surface groups.

**Table 1:** Total carboxyl group concentration (mol/g) inserted into PLLA scaffolds as a function of the reaction time (min).

Material	Hydrolysis time (s) ^#^	Carboxyl group concentration (mol/g)
PLLA	0	(3.7 ± 0.5) × 10^−5^
	20	(5.6 ± 0.9) × 10^−5^
	45	(5.5 ± 1.0) × 10^−5^
	60	(5.8 ± 0.7) × 10^−5^ *
	180	(5.9 ± 1.0) × 10^−5^ *

**^#^** Reaction with 0.1 M NaOH; * Significant difference when compared to PLLA scaffolds without hydrolysis (0 s); *p* < 0.05.

We also observed severe limitations for aminolysis, in which total degradation of the fibers was observed when using conditions reported in literature [24,25]. As shown in Table 2, we observed a reaction time limit of 5 min using diamine concentrations that were 3–8 times lower than previously reported, whereas the amine concentration on the surface varied by only 2.5-fold.

**Table 2:** Total amine group concentration (mol/g) inserted into PLLA scaffolds as a function of reaction time (min).

Material	Aminolysis time (min) ^#^	Amine group concentration (mol/g)
PLLA	0.5	(1.2 ± 0.2) × 10^−5^
	1	(1.6 ± 0.2) × 10^−5^
	3	(1.9 ± 0.2) × 10^−5^ **
	5	(3.2 ± 0.2) × 10^−5^ ***

^#^ Reaction at 0.008 g/mol of HAD; ** Significant difference when compared to PLLA scaffold with 0.5 s of aminolysis at *p* < 0.01 and *** *p* < 0.001.

After aminolysis or hydrolysis, dialdehyde (GTA) and carbodiimide (EDC) chemistry can be respectively employed to immobilize collagen onto the polymer surface [11,20], although some cytotoxicity is attributed to GTA [24].

The amount of collagen incorporated onto the scaffolds’ surface was measured with the ninhydrin test and elemental analyses (Table 3). Using both methods, approximately 1.5% collagen could be inserted via hydrolysis/EDC methodology. However, for the aminolysis/GTA method, the amount of collagen detected by elemental analysis was 5-fold higher than the values determined using the ninhydrin test. Although similar values were observed among hydrolysis/EDC, elemental analyses exhibited high standard deviation and coefficient of variance values. Because the collagen was not well distributed in these materials, the high variability can be attributed to the small amount of material used for analyses. However, the ninhydrin test quantification results, which required more material for analyses, exhibited lower variance; thus, the ninhydrin test was a more efficient quantification methodology for these materials. This observation is in agreement with the higher concentration of functional groups obtained after surface modification, establishing functionalization as the limiting step of this approach.

**Table 3:** Means and standard deviations of collagen (wt%) inserted into the scaffolds obtained from elemental analyses and the ninhydrin test.

Material	Collagen concentration (wt%)	
	Elemental analysis	Ninhydrin test
PLLA	0	-
PC_hydrolysis	1.4 ± 0.5	1.5 ± 0.2
PC_aminolysis	1.5 ± 0.6	0.30 ± 0.05
PC_blend	47 ± 1	-
PC_cf_HFP	71 ± 10	-
PC_cf AA	15 ± 6	-

Co-electrospinning is a technique that offers the advantage of incorporating large amounts of collagen. In addition, because this technique creates segregated fibers of each of the compounds from different solutions, alternative solvents can be selected, allowing a lower extent of collagen denaturation during electrospinning [13,14]. In this study, acetic acid and HFP were compared as solvents that have different effects on collagen denaturation. Collagen is highly stable in these solvents, however, resulting in a high threshold concentration for achieving the viscosity conditions necessary for electrospinning to occur without bead formation.

The co-electrospinning of PLLA and collagen was optimum when using concentrations of 50 wt% polymers. Although initial solutions contained the same concentrations of PLLA and collagen, their distinct properties, such as viscosity, surface tension and charge density, affected their ability to be electrospun [25], hence yielding hybrid mats of varied composition. Elemental analyses (CHN) were performed to determine the final amounts of collagen in the materials (Table 3). The collagen solution formed using HFP was able to incorporate more collagen than the acetic acid solution: the former yielded mats containing 71% collagen, whereas the latter yielded mats containing only 15% collagen.

Finally, PLLA and collagen blends were produced using a co-solvent that equally dissolved both polymers. In this case, HFP was observed as the only solvent in which the two polymers were miscible, despite the loss of collagen’s most important structural features, such as its triple helices assembly [13]. To create a standard for comparison, co-solutions containing 50 wt% of both polymers were electrospun. The resulting blended material exhibited collagen incorporation closest to its initial composition (47% collagen, Table 3).

**Figure 1:**
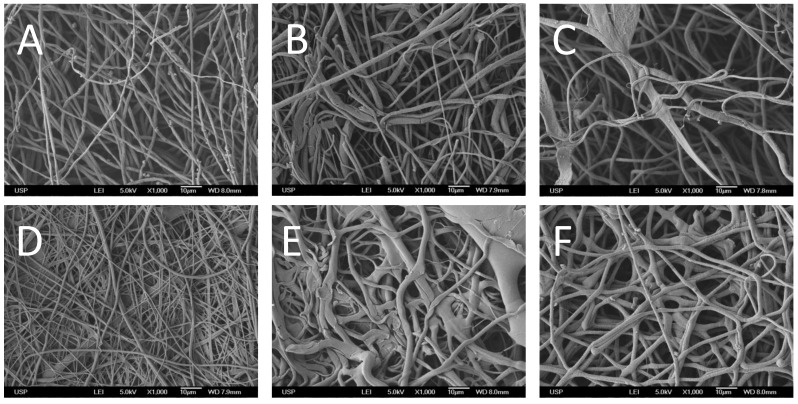
SEM images (×1000 magnification) of electrospun scaffolds: (**A**) PLLA; (**B**) PLLA after hydrolysis and collagen incorporation with EDC; (**C**) PLLA after aminolysis and collagen incorporation with GTA; (**D**) PLLA/collagen electrospun by blending; (**E**) PLLA/collagen electrospun by co-electrospinning and using HFP as the collagen solvent; and (**F**) PLLA/collagen electrospun by co-electrospinning and using acetic acid solution as the collagen solvent.

Figure 1 presents SEM images of the different scaffolds evaluated in this study. When collagen was immobilized on the surface of PLLA through hydrolysis (Figure 1B) or aminolysis (Figure 1C), the fibers became thicker, while a fibrous structure was maintained. For comparison, Figure 1A shows plain PLLA fibers. The scaffold obtained from PLLA/collagen blend electrospinning (Figure 1D) contained regular but thinner fibers, whereas the materials obtained by co-electrospinning clearly consisted of molten fibers, a typical result observed when pure collagen is spun. Nonetheless, the fibers obtained from the co-spinning of the acetic acid/collagen solution were more regular than those obtained using HFP.

Table 4 presents the mechanical scaffold characterization results, a property of utmost importance when evaluating the possible applications of these materials. The materials subjected to hydrolysis and aminolysis, followed by collagen immobilization, exhibited the lowest Young’s modulus and tensile strength values. These findings are somewhat expected because diamines are known to percolate through the polymer matrix, followed by lysis reactions and disruptions of fibers, decreasing the strength and elasticity of the material [26]. Kim and Park [26] reported that PLLA nanofibers subjected to aminolysis developed stacked lamellae through transverse oriented degradation, resulting in lower mechanical properties.

However, basic hydrolysis is expected to be solely a surface reaction. Sun *et al.* [27] demonstrated that alkali etching of PLLA yarns promotes time-dependent mass loss, concluding that the process is surface-limited. This surface peeling causes a reduction in fiber diameter, which is also dependent on alkali concentration and temperature. However, in this study, the electrospun fiber diameters were 2 orders of magnitude lower than those of the yarns, and the same peeling was expected to break down most of the low-diameter fibers, explaining the results reported in Table 4.

**Table 4:** Mechanical properties of PLLA/collagen electrospun materials: means and standard deviations of Young’s modulus ^1^, tensile strength and elongation at break values of the six scaffolds tested.

Material	Elongation at break (%)	Tensile strength (MPa)	Young’s modulus ^1^ (GPa)
PLLA	39 ± 8	18 ± 3	0.19 ± 0.03
PC_hydrolysis	14 ± 2 ***	4.8 ± 0.8 *	0.07 ± 0.01
PC_aminolysis	5.8 ± 0.3 ***	7 ± 1	0.10 ± 0.02
PC_blend	(5 ± 1) × 10 ^1^	(8 ± 1) × 10 ^1^ ***	1.0 ± 0.1 ***
PC_cs_HFP	6 ± 1 ***	38 ± 6 ***	1.4 ± 0.2 ***
PC_cs_AA	14 ± 2 ***	24 ± 4	0.7 ± 0.1 ***

^1^ Young’s modulus is calculated from the linear region of the stress-strain curves; * Significant difference when compared to PLLA scaffold at *p* < 0.05 and *** *p* < 0.001.

Although hydrolysis results in higher scaffold degradation and consequently, lower mechanical properties, the higher surface enables the incorporation of higher amounts of collagen, when compared to aminolysis, as previously discussed.

Regarding DMA analyses of materials with a large amount of collagen obtained by co-electrospinning, the lower elongation of these materials at break is believed to be a function of neat-collagen crosslinked fiber friability. While electrospun collagen fiber elongation has been observed to reach values of 26% and 33% in literature [28,29], these values refer to a scaffold not submitted to any crosslinking treatment; in this present study, materials were analyzed after being crosslinked. Although collagen scaffolds could be obtained for electrospinning, they could not be tested due to their friability after crosslinking treatment. Co-electrospun scaffolds also exhibited higher rigidity, as indicated by their high elastic moduli (Table 4). Previous studies [21,29] have reported that the Young’s moduli of electrospun collagen fibers are related to fiber diameter. Yang *et al.* demonstrated that the Young’s modulus ranged from 1.0 to 3.9 GPa in scaffolds with mean fiber diameters of 187 and 305 nm, respectively. In addition, no significant difference was observed in the Young’s moduli between crosslinked and uncrosslinked collagen fibers. These values are at least 5 times higher compared with those of PLLA examined in this present study, explaining the higher Young’s modulus values obtained in co-electrospun and blended materials.

In the blended materials, the PLLA/collagen scaffold was able to maintain high elongation via PLLA and high tensile strength via collagen. Consequently, this material exhibited better mechanical properties than the other scaffolds evaluated in this study. These results agree with those of Ngiam *et al.* [30] who reported that 1:1 PLLA/collagen scaffold blend exhibited an elasticity modulus that was approximately 3.5 times higher than that of pure PLLA after hydroxyapatite incorporation.

### 2.2. Cell Culture

**Figure 2:**
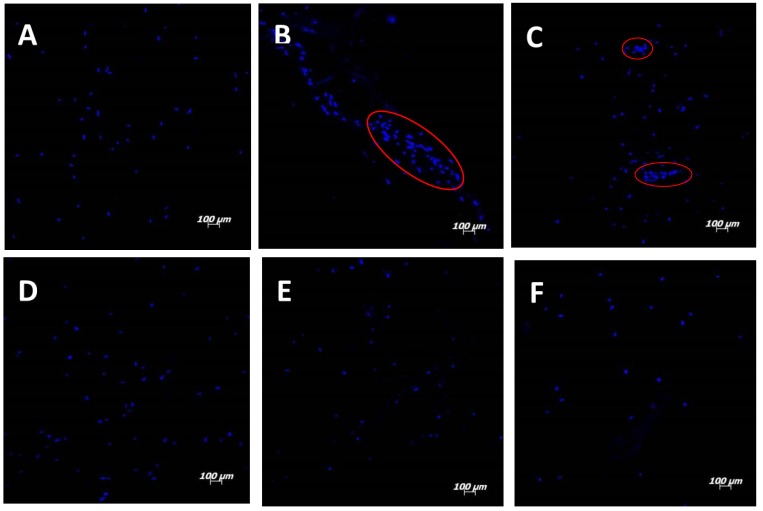
Fluorescence confocal images (100× magnification) of cells on electrospun scaffolds: (**A**) PLLA; (**B**) PLLA after hydrolysis and collagen incorporation with EDC; (**C**) PLLA after aminolysis and collagen incorporation with GTA; (**D**) PLLA/collagen electrospun by blending; (**E**) PLLA/collagen electrospun by co-electrospinning and using HFP as the collagen solvent; and (**F**) PLLA/collagen electrospun by co-electrospinning and using acetic acid solution as the collagen solvent.

Fluorescence images of labeled cell nuclei obtained by confocal microscopy are presented in Figure 2; the cells were homogeneously distributed on top of the majority of the materials, except on the PC_hydrolysis scaffold and to a lower extent on the PC_aminolysis scaffold, which contained regions of concentrated cells (Figure 2B,C). Because small amounts of collagen were bound to the scaffold in these methods, collagen only partially covered the fibers, creating preferential sites for cell adhesion and proliferation. In fact, the collagen concentration increased the number of sites available for integrin and other transmembrane collagen receptor attachment, responsible for cell adhesion and proliferation [31,32]. Likewise, studies have demonstrated that increasing the concentration of immobilized collagen on PLLA/collagen membrane surfaces is correlated with increasing cell density [20,33].

**Figure 3:**
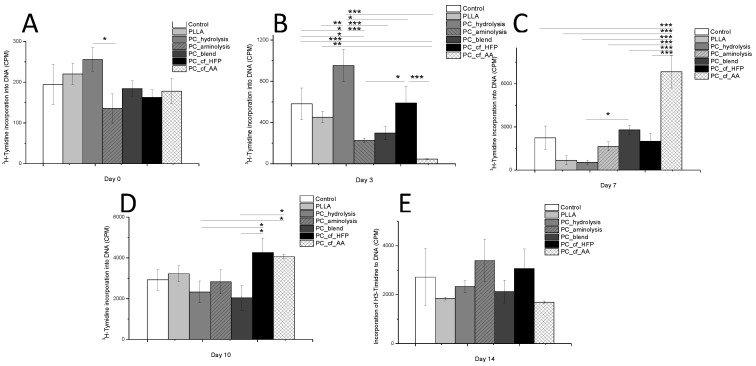
Incorporation of ^3^H-thymidine (CPM) into the DNA of SHEDs after (**A**) 0; (**B**) 3; (**C**) 7; (**D**) 10; and (**E**) 14 days of culture on different PLLA and collagen scaffolds. Statistically significant difference at * *p* < 0.05, ** *p* < 00.01 and *** *p* < 00.001.

Cell proliferation was measured by ^3^H-thymidine incorporation into DNA (Figure 3), and cell differentiation (osteogenesis) was assessed by alkaline phosphatase (ALP) activity (Table 5). The results show a different growth rate for each type of scaffold upon culturing human mesenchymal stem-cells from exfoliated teeth dental pulp (SHEDs) for up to 14 days. Initially, up to day 3, PC_hydrolysis exhibited higher adhesion and proliferation than all of the other materials. One possible explanation for this result is that the collagen attached to this functionalized surface was less degraded than electrospun collagen [21], facilitating cell adhesion [5,16,20] through a highly specialized mechanism of interaction with the collagen triple-helix [32]. Furthermore, hydrolysis increases polymer hydrophilicity, favoring cell attachment and growth [34]. The variation observed between PC_hydrolysis and PC_aminolysis can be attributed to the 5-fold lower content of collagen and the presence of half as many functional groups during PC_aminolysis, limiting the effect of this process.

**Table 5:** ALP activity (U/L) means and standard deviations in SHEDs cultured on scaffolds under ODM and DMEM conditions.

Material	ALP (U/L)	
	ODM	DMEM
PLLA	0.16 ± 0.05	Not detected
PC_hydrolysis	0.60 ± 0.08	0.15 ± 0.04
PC_aminolysis	0.5 ± 0.1	Not detected
PC_blend	1.2 ± 0.1 ***	0.8 ± 0.1
PC_Cf_HFP	1.0 ± 0.2 ***	0.34 ± 0.08 **
PC_cf_AA	1.6 ± 0.3 ***	Not detected
Control	1.3 ± 0.1 ***	Not detected

*** Significant difference of ALP in ODM medium when compared to the PLLA scaffold at *p* < 0.001; ** Significant difference of ALP in DMEM medium when compared to the PLLA/collagen blended material at *p* < 0.01.

From day 7 to 10, higher cell proliferation rates on the co-electrospun materials were observed. These results can be attributed to two main factors, namely, the amount of collagen and cells adhering to cryptic binding sites in collagen, which are only exposed after collagen denaturation [31]. Finally at day 14, advanced cell maturation leads to decreased cell metabolism and equalized growth rates for all groups. These results agree with studies that have demonstrated higher early cell adhesion when collagen was added to scaffolds of various compositions [20,33].

ALP is an enzyme that is used as a biochemical marker of osteoblastic activity. ALP activity was used to assess SHED cell differentiation when plated onto scaffolds and cultured with ODM or DMEM. Osteodifferentiation should be observed on the materials cultured in the presence of ODM, whereas cells that are cultivated in basal medium (DMEM) should differentiate as a function of the scaffold material. Table 5 shows that the materials containing higher amounts of collagen, as obtained by blending or co-electrospinning, exhibited higher expression of ALP in ODM as well as in pure DMEM. These findings can be attributed to the larger number of sites for integrin adhesion, which promote better cell dispersion and improve osteoblast differentiation [35,36]. Integrins are a class of transmembrane proteins known for mediating cell adhesion to the extracellular matrix and consequently, providing an intracellular signaling for cell proliferation, functionality and/or differentiation [35]. Studies performed by Mizuno *et al.* [35] and Schneider *et al.* [36] reported that the Asp-Gly-Glu-Ala domain of type I collagen interacts with α2β1 integrin, increasing gene expression of bone markers and matrix mineralization [35,36].

However, not all materials were able to promote the differentiation of mesenchymal stem cells (SHEDs) into osteoblasts without ODM. This osteodifferentiation ability was observed on only three materials, namely, the scaffolds obtained by co-electrospinning using HFP, by collagen immobilization after hydrolysis and by polymer blending, with the latter exhibiting the highest extent of osteogenic induction. The main but not exclusive factor responsible for osteoblastic differentiation was the presence of collagen on the scaffold because the ALP activity was very low or non-detectable in both pure PLLA or in the control without collagen. These findings agree with those of a previous study that demonstrated gene expression of bone markers in collagen but not in PLLA scaffolds under basal conditions [37].

However, the presence of collagen does not ensure differentiation because the MSCs grown on the PC_aminolysis and PC_cs_AA scaffolds did not exhibit ALP activity in basal medium. Other factors operate in conjunction to determine the osteodifferentiation capacities of a material. Comparing a non-osteoinductive material (PC_aminolysis) with an osteoinductive material (PC_hydrolysis) reveals that the main difference between them is collagen content, indicating that a minimum amount of collagen in the scaffold is required to promote cell differentiation.

When comparing the materials obtained by co-electrospinning, both were observed to contain independent fibers of PLLA and collagen, with the main difference between them being the solvent used to obtain the collagen solution. According to Table 5, collagen dissolved in HFP is able to promote cell differentiation in DMEM, whereas in acetic acid, cell differentiation cannot be promoted. Dong and co-workers [38] demonstrated that HFP solubilizes collagen not only by breaking the hydrophobic interactions via the trifluoromethyl groups but also by breaking hydrogen bonds via the mildly acidic secondary alcohol hydroxyl. Acetic acid would promote solubilization only via hydrogen bond rupture, which may produce differences in the collagen fibers obtained using these methodologies. A study by Liu *et al.* [14] using circular dichroism analysis demonstrated that electrospun scaffolds of collagen obtained with HFP and acetic acid caused collagen denaturation due to the loss of the triple-helix conformation. Moreover, when using acetic acid, the extent of collagen degradation was reduced compared with that observed using HFP [14]. The higher degradation afforded by HFP would easily expose cryptic binding sites to integrin receptors, as previously described [31]. However, partial degradation by acetic acid would induce sterically hindered links to these sites. Therefore, higher bone differentiation could be obtained using HFP.

The highest extent of osteoinduction was observed on the blended material. Several factors that are unique to these materials’ design can interact to induce and improve bone differentiation, such as reduced fiber size, fibers composed of both collagen and PLLA and highest mechanical properties [37,39].

The data obtained in this study allow us to conclude that the material obtained by electrospinning a co-solution of PLLA/collagen yielded the best performance for bone tissue engineering applications because this material demonstrated the highest mechanical properties and high values of ALP expression in osteoinductive and basal media, exhibiting osteoconduction and osteoinduction properties.

## 3. Experimental Section

Five methodologies were designed to compare the mechanisms by which collagen is inserted into scaffolds, namely: (1) immobilization of collagen on the surface of electrospun PLLA subjected to hydrolysis using carbodiimide as the crosslinking agent (PC_hydrolysis); (2) immobilization of collagen on the surface of electrospun PLLA subjected to aminolysis and using glutaraldehyde as the crosslinking agent (PC_aminolysis); (3) electrospinning of a co-solution of PLLA and collagen (1:1) using HFP as co-solvent (PC_blend); (4) co-electrospinning (concomitant spinning of isolated solutions) of PLLA/chloroform and collagen/HFP solutions (1:1) (PC_cs_HFP); (5) co-electrospinning of PLLA/chloroform and collagen/acetic acid solutions (PC_cs_AA).

### 3.1. Electrospinning of PLLA, Collagen and PLLA/Collagen Solutions

A PLLA/collagen blend scaffold in a 1:1 ratio was obtained by the co-dissolution of both polymers in HFP, producing a 5 wt% solution, followed by electrospinning in one syringe.

PLLA/collagen hybrid scaffolds formed in a 1:1 ratio using different solutions were prepared through the simultaneous co-electrospinning of a PLLA solution (5 wt%) in chloroform/DMF (9:1) and a collagen solution (25 wt%) in acetic acid (40 vol%) or a collagen solution (5 wt%) in HFP. Solutions were electrospun using different syringes coupled along the same axis but in different directions and perpendicular to the collector.

Pure PLLA solution (5 wt%) in chloroform/DMF (9:1) was electrospun for further collagen immobilization. All solutions were electrospun using the following parameters: flow rate of 2 mL/h, distance of 9 cm between the needle and collector and applied voltage of 12.5 kV. The collector was a rotor operated at low speed.

### 3.2. Scaffold Crosslinking

Because collagen fibers are partially soluble in aqueous media after electrospinning, the scaffolds obtained using co-spun or blended fibers were stored in a glutaraldehyde (GTA) atmosphere for 24 h to crosslink the scaffold fibers. After crosslinking treatment, the scaffolds were washed four times with a 0.02 M glycine solution for 20 min and once with deionized water to remove and neutralize the remaining GTA.

### 3.3. PLLA Functionalization and Collagen Immobilization on the Surface

PLLA electrospun scaffolds were submitted to aminolysis with 1,6-hexanediamine (HDA) or to hydrolysis to create surface amino and carboxylic groups, respectively.

For aminolysis, the scaffolds were immersed in a propanol solution containing 8 mg/mL of HDA at 50 °C for 5 min. Collagen was immobilized on the scaffold surfaces by immersion for 3 h at room temperature in a solution containing 1 wt% GTA in PBS (pH 7.4), rinsed with deionized water for 2 h and immersed in a collagen solution at 4 °C for 24 h [20].

For hydrolysis, PLLA scaffolds were immersed in an aqueous solution of 0.1 M NaOH at 37 °C for 3 min. A 2 mg/mL collagen solution was prepared in aqueous acetic acid (3 vol%). These scaffolds were then immersed in a 48 mM 1-ethyl-3-(3-dimethylaminopropyl)carbodiimide chloride (EDC) solution, 6 mM *N*-hydroxysuccinimide and 50 mM MES (2-(*N*-morpholino)ethanesulfonic acid) buffer (pH 5.0) for 24 h at 4 °C, rinsed with deionized water for 2 h and immersed in a collagen solution for 24 h at 4 °C [11].

### 3.4. Aminolysis, Hydrolysis and Collagen Quantification

The number of amine groups inserted into the scaffolds was measured using the ninhydrin test. Briefly, scaffolds (*r* = 0.6 mm, approximately 5 mg per disc) were immersed for 45 s in an ethanol solution containing 0.01 M ninhydrin, transferred to a clean glass tube and heated to 70 °C for 10 min. Scaffolds were solubilized in a solution containing dichloromethane and isopropanol (1:1). The absorbance of ninhydrin-amine group complexation was measured at 540 nm and compared with a calibration curve to determine the amine concentration in the scaffolds.

The number of carboxyl groups inserted into the scaffolds was measured using rhodamine-carboxylic acid interaction. Briefly, rhodamine-6G hydrochloride was dissolved in a phosphate buffer (pH 12), followed by toluene extraction. PLLA scaffold discs (*r* = 0.6 mm; approximately 5 mg per disc) were solubilized in 1 mL of dichloromethane, followed by the addition of 1 mL of a neutral rhodamine-6G toluene solution. The solution was incubated in the dark for 1 h. The absorbance of rhodamine-carboxylic acid complexation at 535 nm was measured and compared with a calibration curve to determine the carboxyl concentration in the scaffolds.

The amount of immobilized collagen was measured using the ninhydrin test. Scaffold discs (*r* = 0.6 mm, approximately 5 mg per disc) were hydrolyzed with a 6 M HCl aqueous solution for 24 h at 120 °C under a nitrogen atmosphere. The solution was dried, and a combination of a ninhydrin solution (0.04 M) and 0.1 M citric acid buffer (pH 5.5) was added. The final solution was heated to 70 °C for 10 min and cooled to 4 °C for 5 min. The absorbance of ninhydrin-amine group complexation (amino acid groups from hydrolyzed collagen) was measured at 560 nm and compared with a calibration curve to determine the collagen concentration in the scaffolds.

### 3.5. Scanning Electron Microscopy

To analyze and compare scaffold morphologies, SEM images were obtained using a JEOL FEG 741F field-emission electron microscope (Tokyo, Japan).

### 3.6. Elemental Analyses

CHN analyses on the scaffolds were performed using a Perkin-Elmer Elemental Analyzer model 2400 Series II. Because only collagen molecules contained nitrogen, pure collagen mats were used to calibrate the amount of collagen inserted into the other scaffolds based on the proportion of nitrogen detected in each material.

### 3.7. Dynamic Mechanical Analysis (DMA)

Scaffolds were cut into specimens measuring 30 mm × 7 mm. Scaffold thickness was measured using a micrometer (Mitutoyo, Japan), and the specimens were subjected to a stress-strain test in a TA Instruments Q800 DMA tester (New Castle, PA, USA). Briefly, the specimens were fixed between two clamps, and a pre-load of 0.01 N was applied for 5 min. The temperature was maintained at 30 °C, and a force ramp of 2 N/min was applied. The tensile strength, Young’s modulus and maximum elongation at break were obtained for each material.

### 3.8. Cell Proliferation

A cell suspension (2.0 × 10^4^ cells/well) of human mesenchymal stem-cells from exfoliated teeth dental pulp (SHEDs) was added to a 24-well culture tray coated with scaffold and cultured in osteoblastic differentiation media (ODM), composed of Dulbecco’s modified Eagle’s medium (DMEM), 10% fetal bovine serum, 10 mM β-glycerophosphate disodium salt hydrate, 50 µg/mL l-ascorbic acid and 1% penicillin/streptomycin (10,000 U/mL/10,000 µg/mL). Cell proliferation was determined using ^3^H-thymidine uptake into DNA. Cultures were maintained for 0, 3, 7, 10 and 14 days. At each time point, cells were labeled with ^3^H-thymidine (0.037 MBq/well or 0.5 µCi/well) for 18 h before harvesting. Cultures were washed with PBS twice before the addition of 500 µL of 5% TCA (twice) to remove unincorporated label, and the cells were then lysed in 0.1 N NaOH and 0.1% SDS for 2 h and harvested onto glass fiber filters. The filters containing ^3^H-thymidine-labeled DNA were counted using a Perkin-Elmer liquid scintillation counter. The results are expressed in counts per minute (CPM).

### 3.9. Alkaline Phosphatase Assay (ALP)

Human MSCs from the dental pulp of exfoliated teeth (SHEDs) were cultured on the top of the scaffolds for 14 days. Half of the scaffolds and cells were maintained in ODM, and the other half were cultured in DMEM only. For the ALP assay, cells were lysed in lysis buffer composed of 1% Triton X-100, 0.9% NaCl and 0.5 M Tris (pH 9.0) under agitation for 30 min at 4 °C. Cell suspensions were then maintained in an ultrasonic bath for 10 min and centrifuged at 1500 rpm for 15 min before supernatant collection. ALP activity assays were performed according to the manufacturer’s protocol (Labtest, Monte Claros, Brasil), and the absorbance at 590 nm was measured using a spectrophotometer (Tecan, Infinite 200 PRO, Mannedorf, Switzerland).

### 3.10. Cell Distribution on Scaffolds

The cell distributions on the scaffolds were determined by fluorescent staining with nucleus marker 4’,6-diamidino-2-phenylindole (DAPI) at an excitation wavelength of 358 nm and an emission wavelength of 461 nm. After 14 days of culture in ODM, the scaffolds were rinsed with PBS and immersed in a 14.3 mM DAPI solution for 5 min. The scaffolds were rinsed three times with PBS and mounted in glass slides using Vectashield as the mounting medium. The scaffolds were analyzed using a confocal microscope (Zeiss, LMS 510 META, Jena, Germany).

### 3.11. Statistical Analyses

For each test, data were subjected to one-way ANOVA and Tukey’s test (α = 0.05) while considering homoscedasticity and normality.

## References

[1] Formhals A. (1934). Process and Apparatus for Preparing Artificial Threads. U.S. Patent.

[2] Bhardwaj N., Kundu S.C. (2010). Electrospinning: A fascinating fiber fabrication technique. Biotechnol. Adv..

[3] Hong J.K., Madihally S.V. (2011). Next generation of electrosprayed fibers for tissue regeneration. Tissue Eng. Part B Rev..

[4] Sabir M.I., Xu X.X., Li L. (2009). A review on biodegradable polymeric materials for bone tissue engineering applications. J. Mater. Sci..

[5] Ma Z., Gao C., Gong Y., Ji J., Shen J. (2002). Immobilization of natural macromolecules on poly-l-lactic acid membrane surface in order to improve its cytocompatibility. J. Biomed. Mater. Res..

[6] Van Wachem B., Beugeling T., Feijen J., Bantjes K., Detmers J.P., van Aken W.G. (1985). Interaction of cultured human endothelial cells with polymeric surfaces of different wettabilities. Biomaterials.

[7] Orgel J.P., San Antonio J.D., Antipova O. (2011). Molecular and structural mapping of collagen fibril interactions. Connect. Tissue Res..

[8] Cen L., Liu W., Cui L., Zhang W., Cao Y. (2008). Collagen tissue engineering: Development of novel biomaterials and applications. Pediatr. Res..

[9] Bottino M.C., Thomas V., Schmidt G., Vohra Y.K., Chu T.M., Kowolik M.J., Janowski G.M. (2012). Recent advances in the development of GTR/GBR membranes for periodontal regeneration—A materials perspective. Dent. Mater..

[10] Glowacki J., Mizuno S. (2008). Collagen scaffolds for tissue engineering. Biopolymers.

[11] Zhu Y., Gao C., Liu Y., Shen J. (2004). Endothelial cell functions *in vitro* cultured on poly(l-lactic acid) membranes modified with different methods. J. Biomed. Mater. Res. A.

[12] Yan S., Li X.Q., Liu S.P., Wang H.S., He C.L. (2010). Fabrication and properties of PLLA-gelatin nanofibers by electrospinning. J. Appl. Polym. Sci..

[13] Zeugolis D.I., Khew S.T., Yew E.S., Ekaputra A.K., Tong Y.W., Yung L.Y., Hutmacher D.W., Sheppard C., Raghunath M. (2008). Electro-spinning of pure collagen nano-fibres—Just an expensive way to make gelatin?. Biomaterials.

[14] Liu T., Teng W.K., Chan B.P., Chew S.Y. (2010). Photochemical crosslinked electrospun collagen nanofibers: Synthesis, characterization and neural stem cell interactions. J. Biomed. Mater. Res. A.

[15] Chen J., Chu B., Hsiao B.S. (2006). Mineralization of hydroxyapatite in electrospun nanofibrous poly(l-lactic acid) scaffolds. J. Biomed. Mater. Res. A.

[16] Zhu Y., Gao C., Liu X., He T., Shen J. (2004). Immobilization of biomacromolecules onto aminolyzed poly(l-lactic acid) toward acceleration of endothelium regeneration. Tissue Eng..

[17] Zhang H., Lin C.Y., Hollister S.J. (2009). The interaction between bone marrow stromal cells and RGD-modified three-dimensional porous polycaprolactone scaffolds. Biomaterials.

[18] Cui W., Cheng L., Li H., Zhou Y., Zhang Y., Chang J. (2012). Preparation of hydrophilic poly(l-lactide) electrospun fibrous scaffolds modified with chitosan for enhanced cell biocompatibility. Polymer.

[19] Zhu Y., Leong M.F., Ong W.F., Chan-Park M.B., Chian K.S. (2007). Esophageal epithelium regeneration on fibronectin grafted poly(l-lactide-co-caprolactone) (PLLC) nanofiber scaffold. Biomaterials.

[20] Tan H., Wan L., Wu J., Gao C. (2008). Microscale control over collagen gradient on poly(l-lactide) membrane surface for manipulating chondrocyte distribution. Colloids Surf. B Biointerfaces.

[21] Yang L., Fitie C.F., van der Werf K.O., Bennink M.L., Dijkstra P.J., Feijen J. (2008). Mechanical properties of single electrospun collagen type I fibers. Biomaterials.

[22] Bramfeldt H., Vermette P. (2009). Enhanced smooth muscle cell adhesion and proliferation on protein-modified polycaprolactone-based copolymers. J. Biomed. Mater. Res. A.

[23] Zeng J., Chen X., Liang Q., Xu X., Jing X. (2004). Enzymatic degradation of poly(l-lactide) and poly(epsilon-caprolactone) electrospun fibers. Macromol. Biosci..

[24] Khor E. (1997). Methods for the treatment of collagenous tissues for bioprostheses. Biomaterials.

[25] Kriegel C., Arecchi A., Kit K., McClements D.J., Weiss J. (2008). Fabrication, functionalization, and application of electrospun biopolymer nanofibers. Crit. Rev. Food Sci. Nutr..

[26] Kim T.G., Park T.G. (2008). Biodegradable polymer nanocylinders fabricated by transverse fragmentation of electrospun nanofibers through aminolysis. Macromol. Rapid Commun..

[27] Sun M., Downes S. (2009). Physicochemical characterisation of novel ultra-thin biodegradable scaffolds for peripheral nerve repair. J. Mater. Sci. Mater. Med..

[28] Carlisle C.R., Coulais C., Guthold M. (2010). The mechanical stress-strain properties of single electrospun collagen type I nanofibers. Acta Biomater..

[29] Ji J., Bar-On B., Wagner H.D. (2012). Mechanics of electrospun collagen and hydroxyapatite/collagen nanofibers. J. Mech. Behav. Biomed. Mater..

[30] Ngiam M., Liao S., Patil A.J., Cheng Z., Yang F., Gubler M.J., Ramakrishna S., Chan C.K. (2009). Fabrication of mineralized polymeric nanofibrous composites for bone graft materials. Tissue Eng. Part A.

[31] Heino J. (2007). The collagen family members as cell adhesion proteins. Bioessays.

[32] Leitinger B. (2011). Transmembrane collagen receptors. Annu. Rev. Cell Dev. Biol..

[33] Cai K., Kong T., Wang L., Liu P., Yang W., Chen C. (2010). Regulation of endothelial cells migration on poly(d, l-lactic acid) films immobilized with collagen gradients. Colloids Surf. B Biointerfaces.

[34] Chua K.N., Chai C., Lee P.C., Tang Y.N., Ramakrishna S., Leong K.W., Mao H.Q. (2006). Surface-aminated electrospun nanofibers enhance adhesion and expansion of human umbilical cord blood hematopoietic stem/progenitor cells. Biomaterials.

[35] Mizuno M., Kuboki Y. (2001). Osteoblast-related gene expression of bone marrow cells during the osteoblastic differentiation induced by type I collagen. J. Biochem..

[36] Schneider G.B., Zaharias R., Stanford C. (2001). Osteoblast integrin adhesion and signaling regulate mineralization. J. Dent. Res..

[37] Schofer M.D., Boudriot U., Wack C., Leifeld I., Grabedunkel C., Dersch R., Rudisile M., Wendorff J.H., Greiner A., Paletta J.R. (2009). Influence of nanofibers on the growth and osteogenic differentiation of stem cells: A comparison of biological collagen nanofibers and synthetic plla fibers. J. Mater. Sci. Mater. Med..

[38] Dong B., Arnoult O., Smith M.E., Wnek G.E. (2009). Electrospinning of collagen nanofiber scaffolds from benign solvents. Macromol. Rapid Commun..

[39] Sisson K., Zhang C., Farach-Carson M.C., Chase D.B., Rabolt J.F. (2010). Fiber diameters control osteoblastic cell migration and differentiation in electrospun gelatin. J. Biomed. Mater. Res. A.

